# The impact of floor space allowance and dietary energy level on finishing pigs, from 65 to 120 kg, on growth performance

**DOI:** 10.1093/tas/txad070

**Published:** 2023-07-01

**Authors:** Chloe S Hagen, Beau Peterson, Eric Parr, Jorge Estrada, Gustavo Silva, Laura L Greiner

**Affiliations:** Department of Animal Science, Iowai State University, Ames, IA 50011, USA; Carthage Veterinary Service, Carthage, IL 62361, USA; Carthage Veterinary Service, Carthage, IL 62361, USA; Carthage Veterinary Service, Carthage, IL 62361, USA; Department of Veterinary Diagnostic and Production Animal Medicine, Iowa State University, Ames, IA 50011, USA; Department of Animal Science, Iowai State University, Ames, IA 50011, USA; Department of Animal Science, Iowa State University, Ames, IA 50011, USA

**Keywords:** energy, floor space, swine

## Abstract

The objective of this experiment was to evaluate the impact of lowering floor space allowance in finishing hogs from 65 to 120 kg when fed high- vs. low-energy diets on growth performance. Eighty-eight mixed-sex pens with 24 ± 1 pigs per pen were randomly assigned by weight in a complete block design to one of eight treatments in a 2 × 4 factorial arrangement with two energy levels: low (**LE**, 3267 ± 15 kcal/kg) vs. high (**HE**, 3389 ± 15 kcal/kg) accomplished through fat inclusion; and four floor space allowances: 0.6, 0.63, 0.65, and 0.67 m^2^/pig. Assigned floor space was accomplished by moveable gates in the rear of the pen which were adjusted at each pig removal until the marketing phase. Pen weight was measured at days 0, 29, and 48, with feed disappearance measured at days 29 and 48 to calculate average daily gain (**ADG**), average daily feed intake (**ADFI**), and gain-to-feed ratio (**GF**). Data were analyzed by pen (SAS 9.4, Cary, NC), as repeated measures, with the fixed effects of floor space allowance, dietary energy level, and the interaction between floor space allowance and energy level. For the overall experiment, decreased floor space had no effect (*P* > 0.1) on ADG, ADFI, or GF. Energy had a significant effect (*P* < 0.01) on ADFI (3.17 vs. 3.12 kg for LE and HE, respectively) and GF (0.35 and 0.36 for LE and HE, respectively), and tended to impact (*P* = 0.08) ADG (1.12 vs. 1.13 kg, for LE and HE, respectively). In conclusion, reducing space allowance from 0.67 m^2^ down to 0.6 m^2^ did not affect the growth performance of pigs from 65 to 120 kg. Pigs fed LE consumed more than the HE diets but had generally similar growth and no difference in body weight.

## Introduction

the economic return of pig production is directly related to the input costs vs. revenue. floor space allowance directly impacts the number of animals housed in a facility and thus the throughput and profitability. previous research has shown that decreasing floor space allowance decreases average daily gain (**adg**), average daily feed intake (**adfi**), and has variable impacts on efficiency (brumm and miller, 1996; gonyou and stricklin, 1998). over the past several years, the average market weight of a pig has steadily increased from around 114 kg in 1990 to upwards of 129 kg today (USDA, 2020). The increase in marketing weight is mostly due to efficient feeding programs and genetic improvement. Producers are faced with the challenge of understanding the required space these larger pigs and modern genetics require.

Floor space and pig body weight have been expressed as an allometric expression originally defined by Petherick and Baxter (1981): *A* = *k* × body weight (**BW**) (kg)^0.67^, where *A* is the area (m^2^) per pig, *k* is a pre-determined constant coefficient, and BW in kg converted to a 2-dimensional value. The optimal value for *k* has been argued and discussed in many papers, but the equation suggests that floor space would need to increase as the body weight increases. Gonyou et al. (2006) identified the critical *k* value for finishing pigs to be 0.0336, with *k* values lower than this resulting in production decline. Ekkel et al. (2003) indicated that to achieve normal “social lying behavior,” finishing pigs should be stocked at a level no less than *k* = 0.033. However, it is impractical to change floor space allowance in a production facility after placement. Therefore, floor space generally remains constant throughout the growing period until marketing cuts, where the heaviest pigs within a pen may be sold while allowing the lighter pigs to continue to grow. As the floor space allowance stays constant, the *k* value from the previous equation decreases as the body weight increases in conventional pork production.

Historically, reductions in daily gain in floor space studies are suspected due to decreased feed intake (Gonyou and Stricklin, 1998), with Flohr et al. (2018) reporting increases in feed intake as the critical *k* value increased. Thus, increasing feed density by increasing energy in low floor space circumstances may be a method to compensate. However, most previous studies have confounded floor space with group size by adjusting the stocking density of the pen to achieve the desired floor space, which restricts the ability to evaluate whether reduced performance is due to the increased competition for resources or restrictions in free space.

The highest floor space allocation in this study was accomplished by utilizing stock pens, with a stocking density of 25 pigs, allowing for 0.67 m^2^ of free floor space per pig. Thus, the objective of this experiment was to determine the impact of lowering floor space allowance beyond 0.67 m^2^/pig during the grow-finish phase on growth performance in combination with two dietary energy levels while keeping group size consistent. The hypothesis is that high dietary energy levels can help combat the negative effects of lowering floor space allowance on the growth performance of pigs.

## Materials and Methods

### General

All procedures in this experiment adhered to the guidelines for the ethical and humane use of animals for research and were approved by the Institutional Animal Care and Use Committee at Iowa State University (IACUC# 20-172).

### Facility

Pigs were housed at a commercial wean-to-finish research facility in Western Illinois of the United States (Carthage, IL). The experiment took place from December 2020 to February 2021. Pens were equipped with 1.28-m wide feeders with three head spaces in each pen (Farmweld Inc., Teutopolis, IL). Two gate mounted water cups per pen allowed 12 ± 1 pigs per water source.

### Animals and Experimental Design

Twenty-one hundred and thirty (2,130) barrows and gilts (PIC 1050 × PIC 337, Hendersonville, TN) were sorted into 88 mixed-sex pens within two barns. Pigs were vaccinated against porcine circovirus type 2, *Mycoplasmal hyopneumoniae*, and ileitis after one week of placement at the wean-to-finish facility (Circumvent PCV-M G2 and Porcilis Ileitis, Merck, Kenilworth, NJ). On day 0, pens of 23 to 25 pigs were blocked by starting body weight (65.2 ± 1.9 kg) and were assigned to a randomized complete block design within barn (11 replications). The barrow-to-gilt ratio was recorded for each pen. Within blocks, pens were randomly assigned to 1 of 8 treatments in a 2 × 4 factorial arrangement with two energy levels: low (**LE**, 3267 ± 15 kcal/kg across phases) vs. high (**HE**, 3389 ± 15 kcal/kg across phases); and four floor space allowances: 0.60, 0.63, 0.65, and 0.67 m^2^/pig. Assigned floor space allowance was accomplished by moveable gates in the rear of the pen based on number of pigs. Floor space allowances did not include space occupied by the feeders or watering cups. Gates were adjusted based on the number of pigs in the pen. In the event of pig removals from a pen, the gates were modified within 7 d of the removal to withhold the appropriate floor space with the remaining pigs.

For a duration of 10 wk prior to the start of the experiment, the pigs were housed in the barn and provided with a common commercial diet. Initially, the pens were populated with 25 pigs each. However, some pens had fewer than 25 pigs at the beginning of the experiment due to pig removals. Pens with 23 to 25 pigs were left unaltered, while pens with fewer than 23 pigs underwent mixing with at least 50% new pigs to ensure each pen contained a total of 25 pigs. The pens requiring mixing were grouped together as blocks, and within each barn, one block consisted of pens containing pigs that had been mixed prior to the start of the experiment.

### Dietary Treatments

The energy level was accomplished by adding fat, while keeping soybean meal, dried distillers grain with solubles, and lysine to metabolizable energy (**Lys:ME**) consistent between each treatment for each phase. Diets were formulated (Table 1) to meet or exceed recommendations of the genetic supplier (PIC, 2016) with appropriate vitamin and mineral requirements (NRC, 2012). A feed budget was developed based on the starting BW and the estimated feed efficiency for the size of the pig but was kept the same for both energy levels. Diets were provided in mash form, with ad libitum access to both feed and water. Feed was delivered to each pen through the automated Howema Feed System (Big Dutchman, Vechta, Germany). Due to historic pig aggression within the facility, magnesium oxide was included in the dietary phase 3 diets to alleviate historic side and flank biting (O’Driscoll et al., 2013).

**Table 1.** Ingredient composition of experimental diets (as-fed basis) for diets fed from 65 to 120 kg

Experimental period	Period 1 (days 0 to 29)^6^				Period 2 (days 29 to 48)	
Dietary phase	Phase 1		Phase 2		Phase 3	
Ingredient^1^	Low	High	Low	High	Low	High
Corn	56.64	54.01	65.60	62.98	72.11	69.51
Soybean meal	15.00	15.00	11.50	11.50	10.00	10.00
DDGS^2^	25.00	25.00	20.00	20.00	15.00	15.00
Fat (corn oil)	0.90	3.40	0.65	3.15	0.50	3.00
Calcium carbonate	1.07	1.05	0.97	0.95	0.87	0.84
Salt	0.49	0.49	0.50	0.50	0.50	0.50
l-Lysine HCl	0.43	0.48	0.38	0.42	0.36	0.40
l-Threonine	0.08	0.10	0.07	0.09	0.09	0.11
l-Tryptophan	0.02	0.03	0.02	0.03	0.02	0.03
HMTBa^3^	0.03	0.06	0.00	0.03	0.01	0.04
Monocalcium phosphate	0.19	0.22	0.16	0.19	0.14	0.17
Mg oxide^4^	—	—	—	—	0.25	0.25
Finishing VTM^5^	0.15	0.15	0.15	0.15	0.15	0.15

^1^Diets were in meal form and manufactured at the NSI feed mill (Carthage, IL).

^2^Dried distillers grain with solubles.

^3^2-Hydroxy-4-methylthiobutanoic acid, Novus MHA (St. Charles, MO).

^4^Included due to observed aggression among all treatments.

^5^Vitamin and trace mineral provided: 2,640 IU vitamin A, 880 IU vitamin D, 26.4 IU vitamin E, 2.75 mg vitamin K, 22 µg vitamin B12, 30.8 mg niacin, 17.6 mg pantothenic acid, 5.5 mg riboflavin, 242 mg/kg zinc (zinc sulfate), 242 mg/kg Fe (iron sulfate), 88 mg/kg Mn (manganese sulfate), 26.4 mg/kg Cu (copper sulfate), and 0.66 mg/kg Se (sodium selenite), and 450 phytase units per kilogram of diet. Phytase released 0.12% avP; 0.105% STTD P, and 0.24 total Ca.

^6^Dietary phase 1 provided days 0 to 5 and dietary phase 2 provided days 5 to 29.

Diets were manufactured at Nutritional Services Inc. (Carthage, IL; Table 1). Feed samples were collected at the beginning, middle, and end of each phase for each dietary treatment, and a composite sample was created. Samples were stored at 0 °C for subsequent analysis. Feed samples were sent to Ward Laboratory (Kearney, NE) for proximate and mineral analysis using wet chemistry. Analysis methods by Ward Laboratory were as follows in duplicate: dry matter: method 930.15, AOAC international, 2007; crude protein: method 990.03, AOAC international, 2007; crude fat: method Am-5-04, AOCS, 2013; Ash: method 942.05, AOAC international, 2007; neutral detergent fiber (NDF): Ankom Technology, 2017; minerals: Campbell and Plank, 1991 and Kovar, 2003. Gross energy was determined in duplicate using an isoperibolic bomb calorimeter (model 6200; Parr Instrument Co., Moline, IL). Benzoic acid (6,318 kcal GE/kg) was used as the standard for calibration and was determined to contain 6,314 ± 9 kcal GE/kg.

### Measurements

Pen body was measured at days 0, 29, and 48 for two experimental periods, period 1: days 0 to 29 and period 2: days 29 to 48. Feed disappearance was measured on days 29 and 48. Dietary phases were developed on BW ranges. Pigs reached the top end of dietary phase 1 diets earlier than expected, therefore, dietary phase 1 (days 0 to 5) and two (days 5 to 29) were provided during the experimental period 1, days 0 to 29. Dietary phase 3 was provided during the experimental period 2, days 29 to 48. Measurements were used to calculate ADG, ADFI, and gain-to-feed ratio (**G:F**). Data collection ended when the first pigs were marketed from the pens (day 48), due to constraints in further adjusting floor space gating.

Floor space requirements often use the following equation: Floor space (m^2^) = *k* × BW (kg)^0.67^. Where *k* is a pre-determined optimum coefficient that can calculate the desired floor space for various-sized pigs, and BW^0.67^ represents the surface area of the pig. Since pigs were assigned a floor space allowance that did not change based on body weight, the *k* value was calculated for each treatment using the assigned floor space allowance and ending body weight, pen average, at the end of each period by $k\text{ ​​ ​​ }={\text{m}}^{2}/{\text{BW}}^{0.67}\text{ ​​ ​​ }$ using the assigned m^2^.

### Statistical Analysis

Data were analyzed as a 2 × 4 factorial design using generalized linear mixed model and mixed model methods (Proc Glimmix and Proc Mixed, SAS 9.4, SAS Inst., Cary, NC) with pen as the experimental unit. The statistical model included the fixed effect of floor space allowance, dietary energy level (high vs. low), and the interaction between the two main effects. Block was included as a random effect. Growth parameters were analyzed as repeated measures with the experimental unit of pen for two periods. When fixed effects were a significant source of variation (*P* < 0.05) least squares means were separated using pairwise t-tests (PDIFF option, SAS 9.4, SAS Inst., Cary, NC) with a Tukey Hoc adjustment to account for multiple comparisons. The ratio of barrow to gilts in each pen was recorded at allotment and tested as a covariate, with no significance on the fixed effects, it was removed from the model. Results were considered significant at *P* ≤ 0.05 and considered a trend at *P* > 0.05 and *P* ≤ 0.10. Data were analyzed using the following model:


$$Y_{ijkl}=\text{μ + }{\text{τ}}_{i}+\upsilon_{j}+{\left(\text{τ}+\upsilon \right)}_{ij}+{\text{ρ}}_{k}+e_{ijkl}\;$$

Where *Y*_*ijkl*_ is the observed value for the *l*th pen within the *i*th floor space allowance and the *j*th dietary energy level of the *k*th block; ${\text{τ}}_{i}$ is the fixed effect of the *i*th floor space allowance (*i* = 1 to 4); $\upsilon_{j}$ is the fixed effect of the *j*th energy dietary energy level (*j* = 1 or 2); (τ ∗ υ) is the interaction between the *i*th floor space allowance and the *j*th dietary energy level; ${\text{ρ}}_{k}$ is the random effect of the *k*th block; and $e_{ijkl}\;$ is the associated variance for the model *Y*_*ijkl*_ of the *l*th pen (*l* = 1 to 88). Repeated measures data included the additional fixed effect of experimental period (1 or 2) and the interaction with the main fixed effects.

Testing of normality and homoscedasticity of the studentized residuals was done using the Univariate procedure. Statistical outliers were identified as data points occurring greater than 3 studentized residuals from the model and were excluded from the analysis. Outliers during any periods were removed from cumulative data. Ultimately, eight pens were identified as outliers and deemed separate from the rest of the dataset for cumulative gain-to-feed data, four additional pens were removed from the study due to having inaccurate pen dimensions at the conclusion of the study. Components to the gain-to-feed ratio were removed or the gain-to-feed ratio was removed if the component was greater than 2 studentized residuals and the other component was a true outlier (studentized residual ± 3). Differences in mortality percentage were tested using the offset function in Glimmix with the pigs removed over the number of pigs placed as the variable with the negative binomial option. Means of mortality percentages are reported. Results were considered significant at *P* < 0.05 and a trend at *P* > 0.05 and *P* ≤ 0.10.

## Results

No interactions between space allowance and dietary energy were significant. Therefore, the main effect means are listed and will be discussed. Pigs began the experiment at an average BW of 65.2 ± 1.9 kg and ended at the first market cut (day 48) at an average BW of 118.7 ± 2.5 kg due to restrictions in further adjusting pen dimensions. Experiment pens started with an average of 24.2 ± 0.9 pigs and ended with 23.7 ± 1.2 pigs per pen.

### Analyzed Diets

Nutrient profile, calculated and analyzed, are shown in Table 2. Gross energy (**GE**) and crude fat (**CF**) of high-energy diets were greater than the GE and CF of low-energy diets, as expected. Crude protein (**CP**) of diets varied from the calculated CP. The analyzed calcium was lower, and phosphorus was higher than the calculated values. Thus, the analyzed C:P was slightly lower than calculated.

**Table 2.** Nutrient composition of experimental diets (as-fed basis) for diets fed from 65 to 120 kg

Experimental period	Period 1 (days 0 to 29)								Period 2 (days 29 to 48)			
Dietary phase	Phase 1 (days 0 to 5)				Phase 2 (days 5 to 29)				Phase 3 (days 29 to 48)			
Diet	Low		High		Low		High		Low		High	
Composition^1^^,^^2^	Calc.	Analy.	Calc.	Analy.	Calc.	Analy.	Calc.	Analy.	Calc.	Analy.	Calc.	Analy.
ME, kcal/kg	3,251	—	3,373	—	3,269	—	3,391	—	3,280	—	3,402	—
GE^3^, kcal/kg	—	3,990	—	4,130	—	3,870	—	4,022		3,855		3,967
Crude protein, %	18.17	18.40	18.08	19.30	15.74	16.90	15.64	16.10	14.11	13.70	14.01	14.50
Crude fat, %	5.20	5.80	7.30	7.50	4.80	4.30	6.90	6.90	4.40	4.30	6.60	5.90
Ash, %	4.45	3.94	4.43	3.94	4.01	3.50	3.99	3.41	3.67	3.66	3.65	3.56
NDF, %	12.50	14.50	12.30	15.40	11.50	13.30	11.30	12.20	10.40	10.60	10.20	7.80
Total Ca, %	0.71	—	0.71	—	0.65	—	0.65	—	0.60	—	0.59	—
Avail Ca, %	0.60	0.51	0.60	0.47	0.54	0.51	0.54	0.41	0.49	0.58	0.48	0.37
P, %	0.46	0.51	0.46	0.49	0.41	0.44	0.41	0.45	0.37	0.38	0.37	0.34
Ca:P	1.31	1.00	1.30	0.96	1.31	1.16	1.30	0.91	1.31	1.53	1.30	1.09
Avail. P, %	0.34	—	0.35	—	0.31	—	0.31	—	0.28	—	0.28	—
STTD P, %	0.36	—	0.37	—	0.33	—	0.33	—	0.30	—	0.30	—
Sodium, %	0.26	0.21	0.26	0.20	0.25	0.22	0.25	0.21	0.24	0.25	0.24	0.22
Lysine, %	1.07	—	1.10	—	0.91	—	0.94	—	0.85	—	0.87	—
SID Lysine, %	0.91	—	0.95	—	0.78	—	0.81	—	0.72	—	0.75	—
SID Lys:ME	2.85	—	2.85	—	2.42	—	2.43	—	2.25	—	2.25	—
SID Met:Lys	0.32	—	0.33	—	0.30	—	0.32	—	0.30	—	0.32	—
SID SAA:Lys	0.57	—	0.58	—	0.57	—	0.57	—	0.57	—	0.57	—
SID Trp:Lys	0.18	—	0.18	—	0.18	—	0.18	—	0.17	—	0.17	—
SID Thr:Lys	0.62	—	0.62	—	0.63	—	0.63	—	0.64	—	0.64	—
SID Leu:Lys	1.67	—	1.59	—	1.73	—	1.64	—	1.66	—	1.58	—
SID Iso: Lys	0.63	—	0.60	—	0.63	—	0.60	—	0.60	—	0.57	—
SID Val:Lys	0.72	—	0.69	—	0.73	—	0.70	—	0.70	—	0.67	—

^1^Diets were in meal form and manufactured at the NSI feed mill (Carthage, IL).

^2^Analyses were carried out by Ward Labs (Kearney, NE) using wet chemistry.

^3^Analysis carried out using isoperibolic bomb calorimeter (model 6200, Parr Instrument Co., Moline, IL).

Ca, calcium; P, phosphorus; Calc., calculated nutrients; Analy., analyzed nutrients.

Where during experimental period 1, dietary phases 1 and 2 were fed; and during experimental period 2, dietary phase 3 was fed.

### Pig Performance

**Figure 1.**
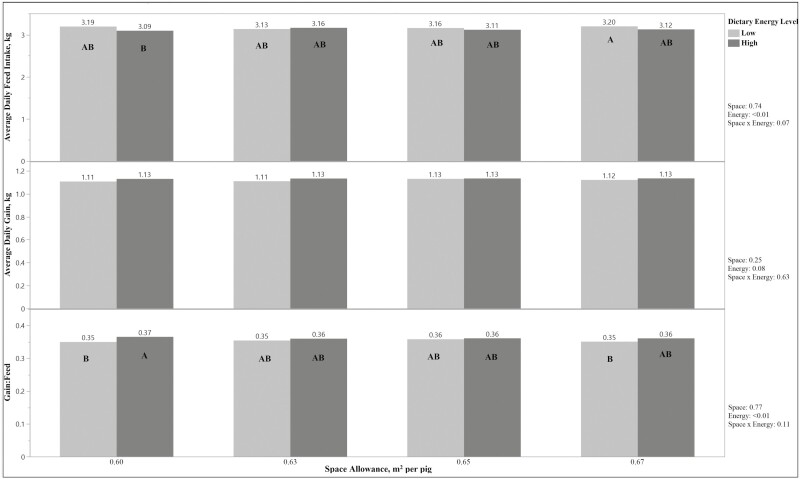
Overall pig performance from 65 to 120 kg for 48-d experiment using four floor space allowances and two dietary energy levels. Axis rows without common letters differ, *P* < 0.05. Values on the right-hand side are *P*-values for each response variable, gain:feed, average daily gain, and average daily feed intake.

Pig performance data from each period and the overall experiment are displayed in Table 3 and Figure 1. Space by period and energy by period tended (*P* < 0.1, Table 3) to impact the body weight measurements. However, there were no statistical differences in pig weight at the end of the experiment. Space by period also impacted (*P* < 0.05) average daily gain, indicating the ADG response to space did not remain consistent between periods. Increased energy level tended (*P* = 0.08) to increase the ADG for the cumulative experiment.

**Table 3.** Main effects of floor space allowance and dietary energy level on body weight (BW), average daily gain (ADG), average daily feed intake (ADFI), gain-to-feed ratio (GF), and mortality percentage of finishing pigs from 65 to 120 kg

	Space allowance, m^2^/pig				SEM	Dietary energy		SEM	*P*-value					
	0.6	0.63	0.65	0.67		Low	High		Space	Energy	Period	Space × energy	Space × period	Energy × period
BW, kg					0.628			0.608	0.42	0.39	<0.01	0.7	0.08	0.096
Day 0	65.16	65.3	65.18	65.29		65.34	65.12							
Day 29	97.05	97.6	97.35	97.82		97.30	97.61							
Day 48	118.37	118.85	119.28	118.69		118.57	119.02							
ADG, kg					0.010			0.008	0.47	0.27	0.47	0.54	0.02	0.3
Days 0 to 29	1.11	1.13	1.12	1.14		1.12	1.14							
Days 29 to 48	1.12	1.11	1.15	1.11		1.12	1.12							
Overall	1.11	1.13	1.13	1.13	0.010	1.12	1.13	0.009	0.25	0.08		0.63		
ADFI, kg					0.021			0.016	0.36	0.02	<0.01	0.09	0.24	0.46
Days 0 to 29	2.97	2.99	2.96	2.99		2.99	2.96							
Days 29 to 48	3.37	3.36	3.42	3.43		3.42	3.36							
Overall	3.13	3.14	3.14	3.16	0.022	3.17^a^	3.12^b^	0.017	0.74	<0.01		0.07		
GF					0.002			0.002	0.65	<0.01	<0.01	0.08	0.12	0.95
Days 0 to 29	0.38	0.38	0.38	0.38		0.37	0.38							
Days 29 to 48	0.33	0.33	0.33	0.32		0.32	0.33							
Overall	0.36	0.36	0.36	0.36	0.003	0.35^b^	0.36^a^	0.002	0.77	<0.01		0.11		
Mortality %^1^	3.90	1.70	1.20	2.38	0.936	2.04	2.15	0.907	0.09	0.88		0.64		

^1^Mortality percentage for overall experiment days 0 to 48.

^a,b^Means without a common superscript differ (*P* ≤ 0.05)

Three-way interaction of space, period, and energy insignificant (*P* > 0.1) for all parameters.

Pigs on the low-energy diet consumed more feed than pigs on the high-energy diet (P < 0.01, Table 3). However, space allowance did not impact (*P* > 0.1, Table 3) feed intake. Space and energy interactions did, however, tend to impact feed intake for each period (*P* = 0.09, Table 3) as well as the overall experiment (*P* = 0.07, Table 3). This indicates a tendency of space to impact ADFI differently at each energy level.

The gain-to-feed ratio was impacted (*P* < 0.01, Table 3) by the energy level of diet. Pigs fed the low-energy diet had a lower G:F than pigs on the high-energy diet. The interaction between space and energy also tended (*P* < 0.1, Figure 1) to impact efficiency due to the impact on feed intake.

The effect of space tended to impact the mortality rate (*P* = 0.09, Table 3). The energy level and the combination of energy and space had no influence on mortality rate (*P* > 0.1, Table 3).

Using the ending body weight at each period and the assigned floor space allowance, the *k* value for each pen at each period was calculated, reported in Table 4.

**Table 4.** Calculated *k* value based on average body weight and assigned floor space allowance

Floor space allowance (m^2^)	0.6	0.63	0.65	0.67
Day 0^1^	0.0365	0.0383	0.0396	0.0407
Day 29^1^	**0.0280**	**0.0293**	**0.0303**	**0.0311**
Day 48^1^	**0.0245**	**0.0257**	**0.0264**	**0.0273**

^1^Calculated using average pen weight of pens with 24 ± 1 pigs, at the end of each experimental period.

Bold type indicated values under critical *k* value, 0.0336 (Gonyou et al., 2006).

*k*-Values calculated using a formula: space per pig (m^2^) = *k* × BW (kg)^0.67^ (Gonyou et al., 2006).

## Discussion

### Floor Space

Initially, all pigs were provided with floor space allowances that were expected to be sufficient and not limit their growth at the start of the experiment. However, as the study progressed, the observed *k* values fell below the threshold that was originally expected to limit growth, even the highest space allocation. The highest space allowance was set at the standard stocking density of the facility used and not designated to be limiting in nature; however, the final *k* value of the highest floor space fell below the critical *k* value of 0.0336 m^2^/BW^0.67^ reported by Gonyou et al. (2006). While we did not see a decrease in performance as the floor space allowances decreased, we can hypothesize the cause was because the group size remained consistent, and the highest floor space may have been restrictive itself. Therefore, further restricting the floor space beyond the highest floor space used in this experiment did not result in growth depression.

Specifically for each 0.001 decrease in *k*, it is expected that ADG will decrease by 0.56% to 1.41%, or an average of 0.98% (Gonyou et al., 2006). The lower floor spaces should have decreased performance beyond the highest floor space allowance in the current study. More recent studies than those analyzed by Gonyou et al. (2006) found reductions in performance at lower body weights than previously predicted (Flohr et al., 2016; Thomas et al., 2017). Additionally, Johnston et al. (2017) suggested that Gonyou et al. (2006) might be underestimating floor space requirements for the current genetics, which result in heavier market weight pigs. As a result, Flohr et al. (2018) developed updated prediction equations that incorporated a database three times larger than the one utilized by Gonyou et al. (2006). These new equations also incorporated larger marketing weights and accounted for the impact of initial and final BW on the proportional increase in space requirement as BW increases.

Using the Flohr et al. (2018) prediction equations which utilize the final *k* value, initial BW, and final BW; ADG for the lowest floor space allowance in this experiment should have been decreased by 3% compared to the highest floor space group. However, the predicted ADG using the equations is notably lower than the ADG observed in this experiment (predicted to be 0.81 and 0.84, for 0.60 and 0.67 m^2^, respectively). Kansas State University adapted the Flohr et al. (2018) equations to adjust performance predictions based on the observed ADG and ADFI within a facility (Kansas State University, 2015), which predicted ADG and ADFI in this experiment to be decreased by 2.2% and 1.5%, respectively (from the highest to the lowest floor space allowance), and G:F predicted to be 0.36 for all treatments using the parameters from this experiment. Shull (2010) summarized that daily gain has historically been reduced by 1.6% to 5.3% per 0.1 m^2^/pig reduction in floor space, and with a 0.07-m^2^/pig difference between the highest and the lowest floor space in this study, a difference of 1.12% to 3.71% in ADG from the highest to the lowest floor space allowance would be predicted. Gonyou et al. (2006) reported a decrease in ADG to an average of 0.98% for each 0.001 decrease in *k*, which would predict a 6.17% decrease in ADG for the highest floor space in the current study compared to a floor space over the critical *k* value and a 2.75% decrease in ADG between the highest and the lowest floor space in this study.

Predictions in performance based on the floor space allowances vary by each research paper; however, the observed changes to lowering floor space are lower than any of the predictions. Ultimately the ADG and ADFI were decreased by 1.76% and 0.95%, respectively, for the overall experiment between the highest and the lowest treatment; however, they were statistically insignificant. The observed G:F of 0.36 for all treatments is consistent with the Floor Space Impact on Pig Performance worksheet (Kansas State University, 2015), which utilized Flohr et al. (2018) equations. Contrary to the predictions made in previous literature, the study’s findings revealed minimal performance changes resulting from the reduction of floor space, indicating a less significant impact than anticipated.

Unique to this study, all floor space allowances assigned were below the critical *k* value of 0.0336, making it challenging to compare to other studies. Peterson (2004) housed 29 pigs per pen at floor spaces of 0.61, 0.68, and 0.74 m^2^/pig (*k *= 0.026, 0.029, and 0.031, respectively), which is a study that utilized similar floor space allowances and methodology to the current study. Peterson (2004) reported a 4.4% reduction in ADG from the highest and lowest floor space (0.61 and 0.74 m^2^) and a 3.4% and 2.2% reduction in ADG and ADFI, respectively, between the lowest two-floor spaces (0.61 and 0.68 m^2^), but no difference in G:F. Again, this is a greater impact of decreasing floor space on ADG and ADFI than observed in the current experiment. However, the ADG was notably lower than in this experiment at 0.93 and 0.90 kg/day for the 0.68 and 0.61 groups, respectively (Peterson, 2004). Carpenter et al. (2018) found a 10% reduction in ADG for pigs from 69 to 83 kg between the 0.91 and 0.63 m^2^ allowances, using both gate adjustment and pig removal, and suggested that growth restriction could begin at *k* values greater than 0.0336 previously reported by Gonyou et al. (2006). Thus, a limitation of the current experiment is that it could not be evaluated whether the highest floor space was restrictive or predict performance at higher space allowances than the facility allowed. It is hypothesized that if logistics allowed, a greater impact of floor space would have been observed if a space allowance was, at minimum, over the *k* value of 0.0336.

Furthermore, it has been shown that housing pigs below the requirements for floor space needed to achieve maximal ADG and ADFI, are the most economical (Powell et al., 1993), as seen in this experiment where all floor space allowances fell below the recommended *k* value. This strategy is utilized frequently within commercial pork production, with housing pigs below requirements and pigs being removed in marketing cuts. The marketing cut strategy removes heavy-weight pigs from a pen to allow smaller pigs extra time to reach the target weight. It has been shown that pigs stocked at a lower stocking density can recover ADG and ADFI during a marketing cut strategy (Flohr et al., 2016). Thus, while pigs at all space allowances in this experiment may have been restricted due to a low floor space allowance, it is unknown if pigs would have compensated during the marketing phase once a proportion of pigs were removed.

The numerical differences in mortality rate in this experiment should be noted due to the economic significance of mortality to the producer. However, results only tended to be impacted by floor space, potentially due to lack of power for mortality data. Peterson (2004) reported increased mortality for pigs housed at 0.61 m^2^ compared to pigs housed at higher levels of 0.68 and 0.74 m^2^. Hamilton et al. (2003) also reported an increase in morbidity and mortality for pigs housed at 0.37 m^2^ from 40 to 80 kg and 0.56 m^2^ from 80 to 120 kg compared to pigs allowed 0.93 m^2^ for the entire period; however, group size was confounded with floor space. While a limitation of the current experiment is the inability to evaluate results in higher floor space allowances, further research on the impact of space allowance on morbidity and mortality is warranted.

### Group Size

A challenge to comparing floor space studies is the often confounding of floor space with group size. It is believed that this experiment did not see growth restriction by decreasing floor space because group size and thus feeder space and water availability stayed the same per group. Many previous studies have confounded floor space with group size by stocking pens with the number of pigs needed to achieve the desired floor space. This method may increase the competition for feed and water compared to those at a higher floor space due to the greater number of pigs in the pen. The goal of using the adjustable gates was to analyze floor space effects without changing the group size of the pen. However, the results in this study do not follow those of previous floor space research, of decreased growth as floor space decreases (Gonyou and Stricklin, 1998).

Johnston et al. (2017) used similar methods of reducing floor space through moveable gates while also keeping group size consistent, and reported that as floor space allowance increased ADG, ADFI, and final body weight also increased. While the Johnston et al. (2017) study used larger floor space allowances of 0.71 up to 1.07 m^2^/pig, in contrast to the floor spaces allowed in our current study, they observed a response that differed from the current study. Once again, potentially due to the floor space allowances utilized in this study having a low range of m^2^/pig and not reaching a high enough floor space allowance.

Although the free space was decreased in the lower space allowances, feeder space, and water availability remained uniform. It is recommended that pigs per feeder space are lower than 12 pigs for maximum growth (Nielsen et al., 1996; Gonyou and Lou, 2000), and water sources are provided for each 20 to 25 pigs (Baxter, 1985), in which all treatments in the current study met. While the current experiment’s data indicated that reducing floor space did not hinder the growth of finishing pigs, the addition of extra pigs to each pen to meet the required floor space allowance instead of using movable gates might have a different effect. Gonyou and Stricklin (1998) found ADG and ADFI to be the same for groups with 15 pigs and two different *k* values of 0.03 and 0.039. While the *k* values were larger than in this study, it further supports that when access to feeders is constant and adequate, the decreasing floor space did not impact growth.

### Dietary Energy

In restrictive floor space studies, the reduction in daily gain is usually due to a decrease in feed intake (Gonyou and Stricklin, 1998), with increases in feed intake as the critical *k* value increases (Flohr et al., 2018). The reduction in feed intake inspired altering the energy density of the two diets used to compensate for the decreased intake. Decreased feed intake in low floor space studies has been hypothesized to be due to obstacles in getting to the feeder, stress, or feed wastage caused by the space allowance (Shull, 2010). However, studies measuring salivary and adrenal gland cortisol levels with similar group sizes (Street and Gonyou, 2008) showed no differences, and studies observing pig behavior at various constant *k* values for space allocation showed little differences (Anil et al., 2007; Street and Gonyou, 2008). Feed wastage from crowded pens cannot be measured when only collecting feed disappearance but is suspected to be a potential cause of varying G:F results from floor space studies due to frequent and interrupted trips to the feeder (Shull, 2010).

Dietary energy density is the first determinant of ADFI (Henry, 1985). Following the pattern of decreased feed intake and improved feed efficiency of pigs provided increasing energy density diets (Beaulieu et al., 2009), the increased feed intake of the lower energy diet in this experiment and improved feed efficiency of the high-energy diets were predicted. As similarly reported by Quiniou and Noblet (2012), with decreasing ADFI as dietary net energy concentration was increased. An objective of this experiment was to evaluate if increasing dietary energy could curb the negative effects of decreasing floor space. While there were trends in space and energy interactions on ADFI and G:F, no negative effects from the space allowance were observed, and this objective was left incomplete. Similarly, it has been found that increasing dietary energy or lysine did not produce any space by diet interactions at floor spaces of 0.56 and 0.78 m^2^/pig (Brumm and Miller, 1996).

## Conclusions

Reduced floor space in this study did not hinder growth performance, unlike many studies that show negative impacts of floor space on the daily gain and feed intake of crowded finishing pigs. First, it can be hypothesized that the pigs at the highest floor space allowance may have already been restricted. Since there was no discernible reduction in growth between the highest and the lowest floor space allocations, it is possible that the highest space allowance in this study was already restrictive, and further decreasing it below the *k* value of 0.027 did not have a significant impact on performance. This suggests that all pigs in the study may have been subject to crowding, potentially explaining the lack of observed differences. Secondly, it could be attributed to consistent group size. Feeder space per pig was equal and unrestricted and did not affect the ability of the pigs to eat and grow, no matter the amount of free space they had in their environment.

The *k* value of 0.027 at the end of the experiment and space allowance of 0.67 m^2^/pig was the highest used in this experiment. Since no reduction in growth was observed between the highest and the lowest floor space, it suggests that possibly the highest space allowance in this study may be restrictive, and further reducing it below the *k* value of 0.027 did not further hinder performance.
